# Hyperprolactinemia and cancer risk: a Swedish population-based cohort study

**DOI:** 10.1530/EC-25-0108

**Published:** 2025-06-19

**Authors:** Christos Himonakos, Louise Emilsson, Sophie Bensing, Katarina Berinder

**Affiliations:** ^1^ Department of Molecular Medicine and Surgery, Karolinska Institutet, Stockholm, Sweden; ^2^ Department of Internal Medicine, Center for Endocrinology and Diabetes, Karlstad Central Hospital, Karlstad, Sweden; ^3^ Department of Medical Epidemiology and Biostatistics, Karolinska Institutet, Stockholm, Sweden; ^4^ Vårdcentralen Nysäter & Centre for Clinical Research, County Council of Värmland, Värmlands Nysäter, Sweden; ^5^ Department of General Practice & General Practice Research Unit (AFE), Institute of Health and Society, University of Oslo, Oslo, Norway; ^6^ Department of Endocrinology, Karolinska University Hospital, Stockholm, Sweden

**Keywords:** hyperprolactinemia, cancer risk, breast cancer, dopamine agonists, prolactinoma

## Abstract

**Objective:**

Prolactin (PRL) promotes cell proliferation, and PRL receptor expression is elevated in various cancer types. However, only a few studies have examined cancer risk in patients with hyperprolactinemia (HPL). The aim of this study was to investigate cancer risk in a nationwide cohort of patients with a diagnosis of HPL, with special emphasis on breast cancer.

**Design:**

In this Swedish population-based cohort study, we used nationwide registries to identify 3,837 patients (2,955 (77%) women) with HPL, treated with dopamine agonists (DA), diagnosed between 2006 and 2019, along with 38,370 controls matched by age, sex, calendar year and county of residence at first HPL diagnosis.

**Methods:**

Cancer outcomes (overall and specific types), as registered in the Swedish Cancer Register, were analyzed using Cox regression, internally stratified by the matching variables and additionally adjusted for diabetes mellitus, obesity, smoking, alcohol overconsumption, hormone replacement therapy and educational level to estimate adjusted hazard ratios (aHRs).

**Results:**

During a median follow-up time of 6.1 years (interquartile range (IQR) 3.4–9.6), 168 (4.6%) new cases of cancer were identified in patients with HPL and 1,608 (4.4%) in the control group (aHR 1.05 (95% CI: 0.89–1.23)). Twenty-eight (0.7%) patients (all women) in the HPL group and 267 (0.7%) in the control group developed breast cancer (aHR 1.02 (95% CI: 0.68–1.51)). Similarly, there was no increased risk of any other site-specific cancer.

**Conclusions:**

In this nationwide cohort study of patients with DA-treated HPL, no increased risk of overall cancer, breast cancer or other site-specific malignancies was observed.

## Introduction

Prolactin (PRL) is predominantly synthesized and secreted by lactotroph cells in the anterior pituitary gland (1). This PRL production is mainly regulated by the inhibitory effect of dopamine, which is secreted by the tuberoinfundibular dopaminergic (TIDA) neurons of the hypothalamus (1, 2). PRL is also secreted to a lesser extent in extra-pituitary tissues and cells, including the mammary gland, placenta, uterus, brain and lymphocytes, where it exerts its effects through autocrine and paracrine mechanisms (1, 3). PRL receptors are found in several human tissues–including the mammary gland, adipocytes, lymphocytes, liver, pancreas, gastrointestinal tract, uterus and ovaries–indicating a contribution of PRL in many physiologic functions (1, 3, 4, 5, 6, 7). The main role of PRL is the promotion of lactation through development of the mammary gland and lactogenesis (1, 8).

Hyperprolactinemia (HPL) is defined as serum PRL levels above the upper limit of the normal reference range (9). The first-line treatment for HPL is pharmacological therapy with a dopamine agonist (DA), which is usually highly effective in normalizing PRL (9, 10).

*In vitro* studies have investigated the potential effects of PRL on various cancer cell types. In rodents, PRL has been shown to enhance the proliferation of breast cancer cells and accelerate mammary carcinoma growth via autocrine or paracrine effects (11, 12). In humans, increased expression of PRL receptors has been observed in breast carcinomas compared to normal breast tissue (13, 14). PRL has also been implicated in enhancing the proliferation of benign prostate cells (15) and certain malignant human prostate cells (16). Furthermore, increased PRL receptor expression has been reported in several cancers, including ovarian and endometrial cancers (17), colorectal cancers (18), and acute myeloid leukemia (19). High levels of locally synthesized PRL have been demonstrated in colorectal cancer (18, 20).

Observational studies in humans have indicated an association between breast cancer risk and circulating PRL levels within the upper normal range (21, 22), although other studies have reported no such association (23, 24). Similarly, prediagnostic circulating PRL levels showed no significant differences in patients with prostate cancer and matched controls (25).

These findings raise the question of whether patients with established HPL have an increased cancer risk. To date, only a limited number of studies have addressed this issue (26, 27, 28, 29, 30, 31). While no increased risk for breast cancer has been observed, findings for other cancer types remain inconclusive (27, 28, 29, 30, 31). The aim of this study was to investigate cancer risk, with special emphasis on breast cancer, in a large cohort of Swedish patients diagnosed with HPL compared to matched reference individuals.

## Materials and methods

### Identification of the HPL cohort

**Figure 1:**
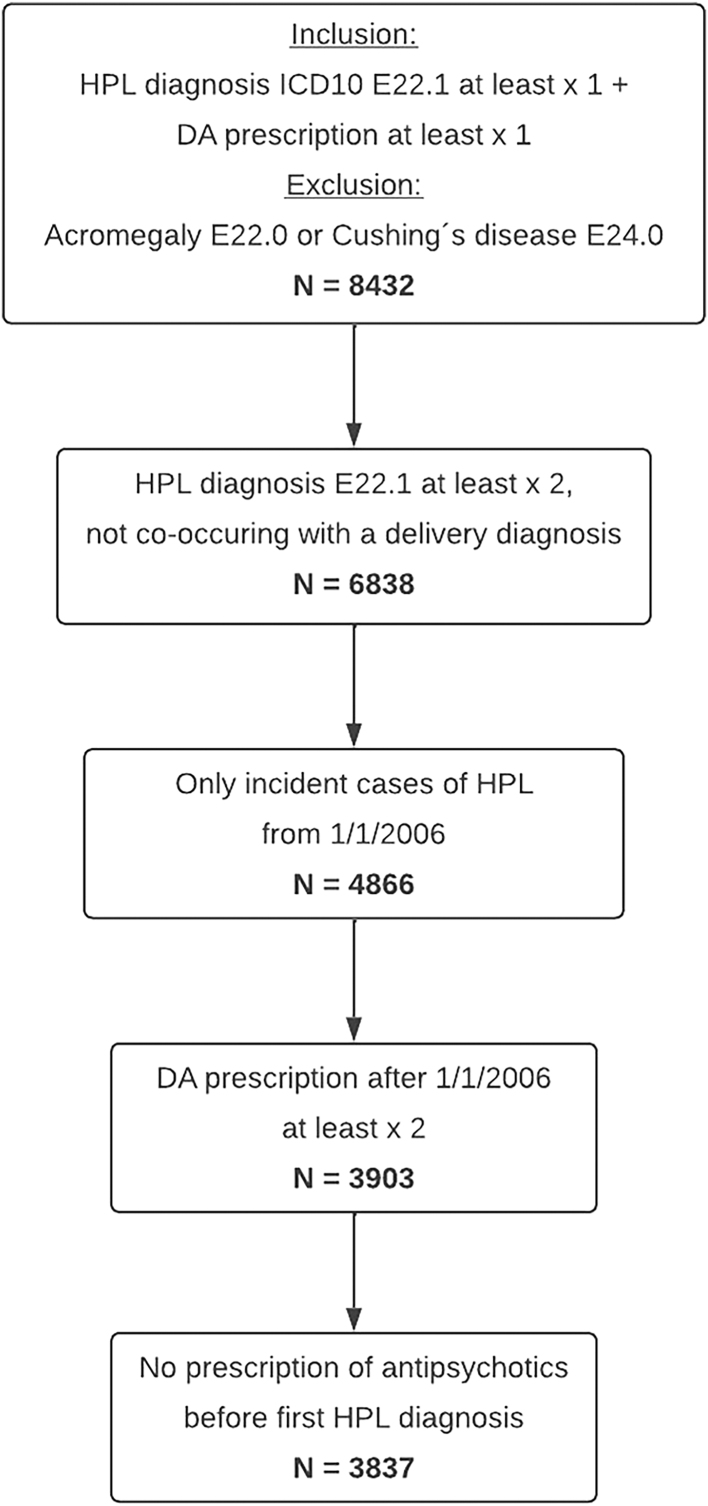
Inclusion of patients with DA-treated hyperprolactinemia. Abbreviations: HPL, hyperprolactinemia; ICD, International Classification of Diseases; DA, dopamine agonist.

A unique personal identification number (PIN) is assigned to all Swedish residents (32). This PIN was used to link all relevant registries. We obtained data from the Swedish National Board of Health and Welfare on all adult individuals (≥18 years) registered with HPL (International Classification of Diseases, 10th Revision (ICD-10) code: E22.1) in the Swedish Patient Registry (33), including both in- and specialized outpatient care. Individuals with a diagnosis of acromegaly (ICD-10: E22.0) or Cushing’s disease (ICD-10: E24.0) were excluded (Fig. 1). In total, we obtained data on 8,432 individuals ever diagnosed with HPL and having at least one prescription of a DA (definitions ATC: G02CB01 or N04BC01 for bromocriptine, G02CB03 or N04BC06 for cabergoline and G02CB04 for quinagolide) registered in the Swedish Prescribed Drug Register (34), from January 1, 2005. We excluded inpatient care registrations related to deliveries (i.e., all discharges including an ICD-10 code: O8X.X, where X could be any number), as these HPL events are more likely to represent peripartum, rather than persistent, HPL. In addition, we requested that the ICD code E22.1 was registered at least twice in the Patient Registry, with the first diagnosis dated on or after January 1, 2006, to ensure the inclusion of incident cases. In total, *n* = 4,866 met those criteria. To further increase the specificity of diagnosing persistent HPL, we also required at least two prescriptions of DAs registered in the Swedish Prescribed Drug Register after January 1, 2006, *n* = 3,903. To further reduce the risk of including cases of drug-induced HPL, we excluded individuals who had any prescription of antipsychotics (ATC: N05A) recorded before their first HPL diagnosis. The final sample of eligible cases consisted of 3,837 individuals (Fig. 1).

### Matched controls

Ten controls for each case (matched for age, sex, calendar year, and county of residence at the time of first HPL diagnosis) were identified by Statistics Sweden using the Total Population Register (35). All Swedish citizens were eligible as controls except individuals previously diagnosed with HPL at the time of matching to the index patient. Our controls represent a random selection of the Swedish population with and without other chronic diseases. Controls were censored from the analyses at the first occurrence of an HPL code as registered in the Swedish Patient Registry.

### Identifying outcome (cancers)

The Swedish Cancer Registry (36) was established in 1958, almost 100% of all malignancies are reported to the Cancer Register and 99% of all cancers are morphologically verified (37). The Cancer Register centrally supplements all different ICD version data (i.e., ICD-8, 9, 10 data) with the corresponding ICD-7 codes. Therefore, we used ICD-7 codes to identify cancers. The following ICD-7 codes were used: any cancer 140–192 and 196–208 (193–195 pituitary gland tumors were not included as they are part of the exposure); oropharyngeal 141–148; gastrointestinal (GI) 150–154 and 159; liver, pancreas, peritoneum 155–158; lung 162; breast 170; female genital 171–176; prostate 177; hematologic cancer 200–208.

### Identifying confounders

Diagnoses on tobacco use (ICD-10: F17, Z71.6, Z72) and/or chronic obstructive pulmonary disease (ICD-10: J43-44) as a proxy for smoking, obesity (ICD-10: E66), alcohol overconsumption (ICD-10: in Supplementary Data (see section on Supplementary materials given at the end of the article)), and diabetes mellitus (ICD-10: E10-14) were collected from the National Patient Register, they were defined as present if any registration before the start of follow-up was available. Furthermore, we also obtained data on highest educational attainment (0–9 years, 10–12 years, >12 years and missing) according to the LISA registry (national database for health insurance and labor market) (38), and any previous prescription of hormone replacement therapy (HRT) in the Swedish Prescribed Drug Register (ATC codes: G03C, G03D, G03F, G03XC and G02BA03).

### Exclusion criteria

Patients with HPL and controls with the relevant outcome registered in the Cancer Register before the start of follow-up were excluded from the study. No other additional exclusion criteria were applied.

### Follow-up

The date of fulfilling all the inclusion criteria, i.e., the date of the second DA prescription or the date of the second HPL diagnosis, whichever occurred last, was set as the start of follow-up for cases and the corresponding date in matched controls. Follow-up ended with death as registered in the Swedish Cause of Death Registry (39), outcome or 31 December 2019, whichever occurred first.

### Statistical analyses

Continuous, non-normally distributed data (age, follow-up time) are presented as the median and interquartile range (IQR). Categorical data are presented as absolute numbers and/or percentages; differences between the groups were evaluated with the Chi-square test. A *P*-value <0.05 was considered statistically significant.

We used internally stratified multivariate Cox regression, comparing each patient to its ten (sex, age, calendar year and county of residence at the time of first HPL diagnosis) matched controls to obtain adjusted hazard ratios (aHRs), hence accounting for the matching variables, and additionally adjusted for diabetes mellitus, obesity, smoking, alcohol overconsumption, HRT and educational attainment. We further calculated the estimates of the adjustment variables on breast cancer risk using multivariate Cox regression. We also performed two separate sensitivity analyses restricted to cases diagnosed at Swedish university clinics and cases diagnosed with benign neoplasm of the pituitary gland (ICD-10: D35.2) because of anticipated higher specificity for persistent HPL. We further ran all outcomes stratified by age (18–45, 46–55, 56+), sex (male, female), calendar year of diagnosis (2006–2010, 2011–2015, 2016–2019) and educational attainment (0–9 years, 10–12 years, >12 years, missing). Analyses were performed using SAS 9.4.

## Results

### Baseline characteristics

A total of 3,837 patients with DA-treated HPL were included in the study, of which 2,955 (77%) were women (Table 1). The median age at fulfilling the inclusion criteria was 34 (IQR 28–43) years and differed between women (median 33 years) and men (median 49 years). Men were more likely than women to have a concurrent diagnosis of a pituitary tumor (26% vs 11%, *P* < 0.001). The patients were evenly distributed across the study enrollment year categories between 2006 and 2019. The initial DA was Bromocriptine in 68% of the patients, Cabergoline in 24% and Quinagolide in 8%. Overall, Bromocriptine was prescribed to 2,822 patients, Cabergoline to 2,169 and Quinagolide to 733 patients at some point during the study period. The median follow-up time was 6.1 (IQR 3.4–9.6) years (Table 1).

**Table 1:** Baseline characteristics of patients with DA-treated hyperprolactinemia.

	Total cohort (*n* = 3,837) *n* (%)	Females (*n* = 2,955) *n* (%)	Males (*n* = 882) *n* (%)
Age (years)	34 (28–43)	33 (27–39)	49 (35–64)
Age categories (years)			
18–44	3,065 (80)	2,675 (91)	390 (44)
45–55	338 (8.8)	183 (6.2)	155 (18)
>55	434 (11)	97 (3.3)	337 (38)
Year of inclusion			
2006–2010	1,200 (31)	963 (33)	237 (27)
2011–2015	1,530 (40)	1,139 (39)	391 (44)
2016–2019	1,107 (29)	853 (29)	254 (29)
Pituitary tumor diagnosis	553 (14)	325 (11)	228 (26)
Initial DA treatment			
Bromocriptine	2,610 (68)	2,107 (71)	503 (57)
Cabergoline	914 (24)	585 (20)	329 (37)
Quinagolide	313 (8.2)	263 (8.9)	50 (5.6)
Follow-up time (years)	6.1 (3.4–9.6)	6.3 (3.5–9.8)	5.8 (3.3–8.8)

Continuous data are presented as the median and interquartile range (Q1–Q3) in parentheses.

Categorical data are presented as numbers (*n*) and percentages (%).

Non-additivity to 100% is due to rounding errors.

Abbreviations: DA, dopamine agonist.

HRT was prescribed more frequently among patients with HPL compared to control subjects (26% vs 18%, *P* < 0.001) (Table 2). There was no statistically significant difference in smoking-related diagnoses between patients with HPL and controls. The differences in other confounding factors including diabetes mellitus, obesity, alcohol overconsumption and educational level are shown in (Table 2).

**Table 2:** Confounders in patients with DA-treated hyperprolactinemia and matched controls at baseline.

	Patients with HPL (*n* = 3,837) *n* (%)	Controls (*n* = 38,370) *n* (%)	*P*-value*
Diabetes mellitus	115 (3.0)	672 (1.8)	<0.001
Type 1	34 (0.9)	245 (0.6)	
Type 2	76 (1.9)	370 (1.0)	
Other/non-specified	5 (0.1)	57 (0.1)	
Obesity	133 (3.6)	997 (2.6)	0.002
Smoking/COPD	30 (0.8)	419 (1.1)	0.074
Alcohol overconsumption	51 (1.3)	955 (2.5)	<0.001
HRT prescription	1,001 (26)	6,993 (18)	<0.001
Educational level			<0.001
0–9 years	449 (12)	4,272 (11)	
10–12 years	1,290 (34)	14,614 (38)	
>12 years	2,066 (54)	18,958 (49)	
Missing data	32 (0.8)	526 (1.4)	
Follow-up time			0.94
<1 year	142 (3.7)	1,465 (3.8)	
1–5 years	1,322 (34)	13,222 (34)	
>5 years	2,373 (62)	23,683 (62)	

* *P* < 0.05 was considered significant.

Categorical data are presented as numbers (*n*) and percentages (%).

Non-additivity to 100% is due to rounding errors.

Abbreviations: DA, dopamine agonist; COPD, chronic obstructive pulmonary disease; HRT, hormone replacement therapy.

ICD-10 codes: diabetes mellitus, ICD-10 codes: diabetes mellitus, E10-14; obesity, E66; smoking, F17, Z71.6, Z72; chronic obstructive pulmonary disease, J43-44; alcohol overconsumption, E24.4, F10, G62.1, I42.6, K29.2, G31.2, G71.2, K70, K85.2, K86.0, O35.4, T51.0, T51.9, R78.0, Y57.3, X65, Y90-91, Z50.2, Z71.4, Z72.1. ATC codes: HRT prescription, G03C, G03D, G03F, G03XC, G02BA03.

### Cancer risk

**Figure 2:**
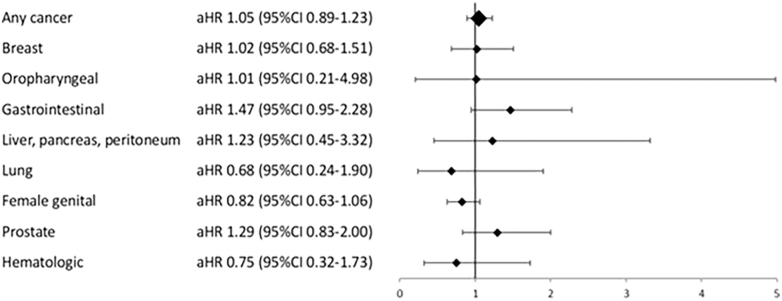
Cancer risk in patients with DA-treated hyperprolactinemia. Abbreviations: DA, dopamine agonist; aHR, adjusted hazard ratios. ICD-7 codes: any cancer, 140–192, 196–208; breast, 170; oropharyngeal, 141–148; gastrointestinal, 150–154, 159; liver, pancreas, peritoneum, 155–158; lung, 162; female genital, 171–176; prostate, 177; hematologic, 200–208.

During the study period, a total of 168 (4.6%) new cases of cancer were identified in patients with HPL compared to 1,608 (4.4%) in the control group (aHR 1.05 (95% CI: 0.89–1.23)) (Fig. 2, Table 3).

**Table 3:** Overall cancer risk in patients with hyperprolactinemia.

	HPL *n* (%)	Controls *n* (%)	Cancer events HPL, *n* (%)	Cancer events controls, *n* (%)	Follow-up years HPL	Follow-up years controls	IR HPL (95% CI)	IR controls (95% CI)	aHR (95%CI)
All	3,638 (100)	36,494 (100)	168 (4.6)	1,608 (4.4)	23,849	239,344	7.0 (6.0–8.1)	6.7 (6.4–7.0)	1.05 (0.89–1.23)
Females	2,832 (78)	28,202 (77)	112 (4.0)	1,127 (4.0)	18,884	187,671	5.9 (4.8–7.0)	6.0 (5.7–6.4)	0.98 (0.80–1.19)
Males	806 (22)	8,292 (23)	56 (6.9)	481 (5.8)	4,964	51,672	11.3 (8.3–14.2)	9.3 (8.5–10.1)	1.24 (0.94–1.65)
Age at enrollment									
18–45 years	2,969 (82)	29,609 (81)	101 (3.4)	995 (3.4)	19,539	194,964	5.2 (4.2–6.2)	5.1 (4.8–5.4)	1.01 (0.82–1.24)
46–55 years	313 (8.6)	3,208 (8.8)	11 (3.5)	155 (4.8)	2,118	21,425	5.2 (2.1–8.3)	7.2 (6.1–8.4)	0.70 (0.38–1.31)
>55 years	356 (9.8)	3,677 (10)	56 (16)	458 (13)	2,190	22,954	25.6 (18.9–32.3)	20.0 (18.1–21.8)	1.30 (0.97–1.72)
Enrollment year									
2006–2010	1,149 (32)	11,505 (32)	81 (7.0)	758 (6.6)	12,489	125,213	6.5 (5.1–7.9)	6.1 (5.6–6.5)	1.08 (0.86–1.36)
2011–2015	1,450 (40)	14,529 (40)	75 (5.2)	667 (4.6)	8,932	89,785	8.4 (6.5–10.3)	7.4 (6.9–8.0)	1.12 (0.88–1.43)
2016–2019	1,039 (29)	10,460 (29)	12 (1.2)	183 (1.7)	2,427	24,344	4.9 (2.1–7.7)	7.5 (6.4–8.6)	0.64 (0.36–1.16)
University hospital	1,524 (42)	15,409 (42)	68 (4.5)	680 (4.4)	10,456	105,852	6.5 (5.0–8.0)	6.4 (5.9–6.9)	0.98 (0.76–1.26)
Pituitary tumor diagnosis	517 (14)	5,160 (14)	32 (6.2)	328 (6.4)	3,545	35,258	9.0 (5.9–12.2)	9.3 (8.3–10.3)	0.99 (0.69–1.43)
Educational level									
0–9 years	405 (11)	3,956 (11)	28 (6.9)	233 (5.9)	2,532	25,966	11.1 (7.0–15.2)	9.0 (7.8–10.1)	1.66 (0.95–2.91)
10–12 years	1,222 (34)	13,859 (38)	55 (4.5)	653 (4.7)	7,933	91,329	6.9 (5.1–8.8)	7.1 (6.6–7.7)	0.97 (0.71–1.32)
>12 years	1,980 (54)	18,164 (50)	83 (4.2)	716 (3.9)	13,205	119,100	6.3 (4.9–7.6)	6.0 (5.6–6.5)	0.99 (0.77–1.27)

Categorical data are presented as numbers (*n*) and percentages (%). Non-additivity to 100% is due to rounding errors.

Abbreviations: HPL, hyperprolactinemia; IR, incidence rate per 1,000 person-years; aHR, adjusted hazard ratio.

Analyses run in strata of cases and ten (sex, age, calendar year and county of residence) matched controls, additionally adjusted for diabetes mellitus, obesity, smoking, alcohol overconsumption, hormone replacement therapy and educational level.

Breast cancer occurred in 28 (0.7%) patients in the HPL group and 267 (0.7%) in the control group, all females (aHR 1.02 (95% CI: 0.68–1.51)) (Fig. 2, Table 4). No statistically significant difference in breast cancer risk was found between the patients and controls after stratification for age categories, enrollment year period or educational level. In addition, no difference in breast cancer risk was observed in women with HPL followed up in a university hospital or additionally registered with a pituitary tumor diagnosis compared to controls (Table 4). The impact of the confounders on breast cancer risk was assessed using multivariate Cox regression analysis (Supplementary Table 1).

**Table 4:** Breast cancer risk in patients with hyperprolactinemia.

	HPL *n* (%)	Controls *n* (%)	Cancer events HPL, *n* (%)	Cancer events controls, *n* (%)	Follow-up years HPL	Follow-up years controls	IR HPL (95% CI)	IR controls (95% CI)	aHR (95%CI)
All	3,835 (100)	38,260 (100)	28 (0.7)	267 (0.7)	25,676	255,709	1.1 (0.7–1.5)	1.0 (0.9–1.2)	1.02 (0.68–1.51)
Females	2,953 (77)	29,445 (77)	28 (0.9)	267 (0.9)	19,985	198,827	1.4 (0.9–1.9)	1.3 (1.2–1.5)	1.02 (0.68–1.51)
Males	882 (23)	8,815 (23)	0	0	5,690	56,882	0	0	-
Age at enrollment									
18–45 years	3,071 (80)	30,601 (80)	22 (0.7)	173 (0.6)	20,532	204,346	1.1 (0.6–1.5)	0.8 (0.7–1.0)	1.19 (0.76–1.88)
46–55 years	336 (8.8)	3,361 (8.8)	3 (0.9)	41 (1.2)	2,292	22,894	1.3 (0.0–2.8)	1.8 (1.2–2.3)	0.81 (0.24–2.73)
>55 years	428 (11)	4,298 (11)	3 (0.7)	53 (1.2)	2,851	28,468	1.1 (0.0–2.2)	1.9 (1.4–2.4)	0.71 (0.22–2.31)
Enrollment year									
2006–2010	1,204 (31)	11,983 (31)	17 (1.4)	146 (1.2)	13,460	133,726	1.3 (0.7–1.9)	1.1 (0.9–1.3)	1.01 (0.60–1.71)
2011–2015	1,527 (40)	15,268 (40)	10 (0.7)	97 (0.6)	9,621	96,139	1.0 (0.4–1.7)	1.0 (0.8–1.2)	1.18 (0.61–2.29)
2016–2019	1,104 (29)	11,009 (29)	1 (0.1)	24 (0.2)	2,594	25,843	0.4 (0.0–1.1)	0.9 (0.6–1.3)	0.35 (0.04–2.67)
University hospital	1,611 (42)	16,095 (42)	8 (0.5)	94 (0.6)	11,297	112,745	0.7 (0.2–1.2)	0.8 (0.7–1.0)	0.95 (0.46–1.96)
Pituitary tumor diagnosis	551 (14)	5,515 (14)	3 (0.5)	46 (0.8)	3,873	38,716	0.8 (0.0–1.7)	1.2 (0.8–1.5)	0.98 (0.29–3.25)
Educational level									
0–9 years	447 (12)	4,262 (11)	8 (1.8)	53 (1.2)	2,919	28,785	2.7 (0.8–4.6)	1.8 (1.3–2.3)	0.97 (0.30–3.11)
10–12 years	1,291 (34)	14,573 (38)	6 (0.5)	109 (0.7)	8,558	98,027	0.7 (0.1–1.3)	1.1 (0.9–1.3)	0.51 (0.21–1.27)
>12 years	2,065 (54)	18,900 (49)	14 (0.7)	105 (0.6)	14,013	125,842	1.0 (0.5–1.5)	0.8 (0.7–1.0)	1.14 (0.62–2.08)

Categorical data are presented as numbers (*n*) and percentages (%). Non-additivity to 100% is due to rounding errors.

Abbreviations: HPL, hyperprolactinemia; IR, incidence rate per 1,000 person-years; aHR, adjusted hazard ratio.

Analyses run in strata of cases and ten (sex, age, calendar year and county of residence) matched controls, additionally adjusted for diabetes mellitus, obesity, smoking, alcohol overconsumption, hormone replacement therapy and educational level.

Regarding other site-specific malignancies, 24 (0.6%) cases of GI malignancies were identified in patients with HPL and 161 (0.4%) in the control group (aHR 1.47 (95% CI: 0.95–2.28)) (Table 5). After stratification for sex, 11 cases (0.4%) of GI malignancies were observed in women with HPL and 53 cases (0.2%) in the control group (aHR 2.12 (95% CI: 1.09–4.11)). In women with HPL there was one case of ventricle cancer (ICD-7: 151), seven cases of colon cancer (ICD-7: 153) and three cases of rectum cancer (ICD-7: 154), while in the control group there were three cases of ventricle cancer, 32 cases of colon cancer, 15 cases of rectum cancer and three cases of small intestine cancer (ICD-7: 152). The risk of GI cancer in men with HPL was not increased (aHR 1.22 (95% CI: 0.68–2.20)). There was no difference in risk for other site-specific malignancies, including oropharyngeal, liver, pancreas, peritoneum, lung, female genital, prostate or hematologic malignancies, between patients with HPL and the control subjects in the whole cohort (Fig. 2, Table 5) or after stratification for sex.

**Table 5:** Overall and site-specific cancer risk in patients with hyperprolactinemia.

Cancer type	HPL *n* (%)	Controls *n* (%)	Cancer events HPL, *n* (%)	Cancer events controls, *n* (%)	Follow-up years HPL	Follow-up years controls	IR HPL (95% CI)	IR controls (95% CI)	aHR (95%CI)
Any cancer	3,638 (100)	36,494 (100)	168 (4.6)	1,608 (4.4)	23,849	239,344	7.0 (6.0–8.1)	6.7 (6.4–7.0)	1.05 (0.89–1.23)
Breast	3,835 (100)	38,260 (100)	28 (0.7)	267 (0.7)	25,676	255,709	1.1 (0.7–1.5)	1.0 (0.9–1.2)	1.02 (0.68–1.51)
Oropharyngeal	3,835 (100)	38,321 (100)	2 (0.1)	19 (0.0)	25,736	256,929	0.1 (0.0–0.2)	0.1 (0.0–0.1)	1.01 (0.21–4.98)
Gastrointestinal	3,816 (100)	38,228 (100)	24 (0.6)	161 (0.4)	25,512	255,786	0.9 (0.6–1.3)	0.6 (0.5–0.7)	1.47 (0.95–2.28)
Liver, pancreas, peritoneum	3,835 (100)	38,326 (100)	5 (0.1)	40 (0.1)	25,730	256,894	0.2 (0.0–0.4)	0.2 (0.1–0.2)	1.23 (0.45–3.32)
Lung	3,833 (100)	38,321 (100)	4 (0.1)	59 (0.2)	25,718	256,774	0.2 (0.0–0.3)	0.2 (0.2–0.3)	0.68 (0.24–1.90)
Female genital	2,873 (100)	28,575 (100)	62 (2.2)	764 (2.7)	19,314	191,522	3.3 (2.5–4.1)	4.0 (3.7–4.3)	0.82 (0.63–1.06)
Prostate	866 (100)	8,605 (100)	23 (2.7)	175 (2.0)	5,473	54,882	4.2 (2.5–5.9)	3.2 (2.7–3.7)	1.29 (0.83–2.00)
Hematologic	3,820 (100)	38,241 (100)	6 (0.2)	79 (0.2)	25,633	256,052	0.2 (0.0–0.4)	0.3 (0.2–0.4)	0.75 (0.32–1.73)

Categorical data are presented as numbers (*n*) and percentages (%).

Abbreviations: HPL, hyperprolactinemia; IR, incidence rate per 1,000 person-years; aHR, adjusted hazard ratio.

Analyses run in strata of cases and ten (sex, age, calendar year and county of residence) matched controls, additionally adjusted for diabetes mellitus, obesity, smoking, alcohol overconsumption, hormone replacement therapy and educational level.

ICD-7 codes: any cancer, 140–192, 196–208; breast, 170; oropharyngeal, 141–148; gastrointestinal, 150–154, 159; liver, pancreas, peritoneum, 155–158; lung, 162; female genital, 171–176; prostate, 177; hematologic, 200–208.

## Discussion

In this large nationwide cohort study of 3,837 patients with DA-treated HPL, no increased risk of overall cancer was observed compared to matched controls from the general population. In addition, no elevated risk of breast cancer or any other site-specific cancer was identified in the overall cohort. The potential cancer risk in patients with HPL is incompletely elucidated. Our findings align with two studies that also reported no increased overall cancer risk in patients with HPL (26, 30), whereas another study found a slight increase (29).

Of special interest is the relation between HPL and breast cancer. There are several case reports of breast cancer in both males and females with prolactinomas (40, 41, 42, 43). On the other hand, treatment with the PRL receptor antagonist LFA102 in monotherapy did not show antitumor activity in 34 patients with metastatic breast cancer (44). In our study, we found no increased risk of breast cancer compared to matched reference individuals. This is in accordance with findings from the previous smaller observational studies of patients with HPL (27, 28, 29, 31). Dekkers *et al.* reported no increased risk of breast cancer in 1,342 women with DA-treated HPL (27). Another register-based study of 2,457 patients (2,130 women and 327 men) with an HPL diagnosis found no increased breast cancer risk (28). Both studies compared the observed breast cancer incidence to the expected incidence in the general population rather than to matched controls (27, 28). In addition, a study of 969 patients (668 women and 301 men) with HPL found no increased breast cancer risk when compared to controls matched by sex, age and county of residence (29). A recent case–control study by Dery *et al.* including 1,484 women with DA-treated HPL reported no increased risk of breast cancer in patients with HPL compared to controls matched for age, sex, BMI and socioeconomic status (31).

In the current study, we adjusted for several risk factors for breast cancer, including smoking, alcohol overconsumption (45) and use of HRT. The Million Women Study showed an increased risk for breast cancer in women on HRT (aRR 1.66 (95% CI: 1.58–1.75)), containing estrogen only (1.30 (1.21–1.40)) or in combination with progesterone (2.00 (1.88–2.12)) (46). In our cohort, more patients with HPL were on HRT compared to controls (26% vs 18%, *P* < 0.001), and thus it was essential to adjust for this confounder when estimating breast cancer risk. Prolonged exposure to endogenous estrogens is also a well-established risk factor for breast cancer, with early menarche, late menopause and nulliparity carrying the largest risk (47, 48). In our study, we lacked information on menstrual and reproductive history, but it is well known that HPL frequently leads to hypogonadism and amenorrhea (2), which may theoretically reduce the risk of breast cancer. Conversely, it is shown that women with HPL have reduced parity, including increased nulliparity (49), which may contribute to an increased breast cancer risk.

In our cohort, all HPL cases were treated with DA at inclusion. Since DA is an effective treatment for HPL (9, 10), normalization of PRL levels during DA treatment may theoretically have counteracted an oncogenic effect of HPL before treatment. However, treatment with ergot-derived DAs (including BRC and CAB) in Parkinson’s disease has been associated with increased overall cancer risk (aOR 2.16 (95% CI: 1.55–2.99)), primarily due to liver cancer, while breast cancer risk is not affected (50). The impact of DA on breast cancer risk is unknown, and the treatment results with DA to patients with breast cancer are contradictory. A study of 70 patients with metastatic breast cancer reported beneficial effects of DA therapy when combined with other chemotherapeutic agents (51). In contrast, a double-blind study of 171 patients with advanced breast cancer found no additional effect of DA to standard treatment (52). International guidelines do not recommend DA for the treatment of either early-stage or advanced breast cancer (53, 54). Given the above, it is unlikely that DA treatment *per se* significantly affected the incidence of breast cancer in our cohort.

Regarding other site-specific malignancies, analogous to breast cancer, we found no increased risk in the total cohort. However, when data were analyzed by sex, an increased incidence of GI malignancies was observed in women with HPL, with 11 cases (0.4%) including ventricle, colon and rectal cancer, compared to 53 cases (0.2%) in the control group. Given the low number of cases and the lack of increased incidence of these cancer types in other studies (29), the above findings are more likely a type I error due to multiple testing rather than an actual increased risk of GI malignancies. The expression of PRL receptors and locally synthesized PRL has been reported in some cases of colorectal cancer (18, 20), while other studies could not confirm these findings (55, 56). Circulating PRL can be higher in patients with colorectal cancer compared to controls and occasionally above the reference range (20, 57, 58) and PRL has been proposed as a prognostic factor in colorectal cancer due to its possible association with a more unfavorable prognosis (57, 58). However, other studies did not find evidence of HPL in colorectal cancer or a correlation between peripheral PRL levels and disease severity (56, 59). Regarding other GI malignancies, a study of 244 patients found no association between prediagnostic PRL levels and esophageal adenocarcinoma (60).

The main strengths of our study are the large size of the cohort and the consistency in follow-up. To the best of our knowledge, this is the largest cohort to date investigating the association between HPL and cancer risk. Furthermore, the Swedish Cancer Registry has almost complete coverage and, combined with the use of the National Patient Register, we could follow the cases and the controls with no loss of follow-up. Limitations to our study are the lack of clinical information and validation of the HPL diagnosis. However, to enhance diagnostic specificity, we combined the Patient Registry and Drug Prescription Registry, including only HPL patients with at least two dispensations of DA to identify persistent cases. Furthermore, the aim of this study was to assess the effect of HPL on cancer risk, irrespective of the etiology of the elevated PRL levels. Another limitation is that we lack information on PRL levels, both before and during treatment. Consequently, we were not able to determine whether cancer risk differs between patients with persistently elevated PRL levels and those with normalized levels, or if there is a correlation between the degree of PRL elevation and cancer risk. However, to potentially identify patients with a higher PRL concentration, we separately examined patients with a concurrent diagnosis of pituitary adenoma and those managed at a university hospital. Nevertheless, we observed no difference in outcomes, suggesting no association. Furthermore, we did not have access to detailed information on treatment duration and adherence to DA therapy. Finally, although we adjusted for several confounding factors, we lacked information on other potential confounders for cancer such as family history and reproductive history.

## Conclusions

In this comprehensive nationwide cohort study of over 3,800 patients with DA-treated HPL, we investigated the potential association between HPL and cancer risk with special emphasis on breast cancer. Our findings revealed no increased risk of overall cancer, breast cancer or other site-specific malignancies compared to matched controls from the general population. Although this study represents the largest investigation to date, further research in large cohorts with comprehensive clinical information is warranted to fully elucidate this relationship.

## Supplementary materials



## Declaration of interest

CH, LE, SB and KB declare that there is no conflict of interest that could be perceived as prejudicing the impartiality of the work reported.

## Funding

This work did not receive any specific grant from any funding agency in the public, commercial or not-for-profit sector.

## Author contribution statement

All authors contributed to the study design. KB and LE supervised the study. LE had full access to data in the study and performed the statistical analysis. CH and KB drafted the manuscript and all authors revised it. All authors approved the final manuscript.

## Data availability

The datasets generated and/or analyzed during the current study are available from the corresponding author on reasonable request.

## Ethics

This study was performed in line with the principles of the Declaration of Helsinki and was approved by the Swedish Ethical Review Authority (Dnr 2019-05232 and Dnr 2024-01811-02).
