# Supplementary material for: Hyperprolactinemia and cancer risk: a Swedish population-based cohort study
Source: Endocr Connect. 2025 Jun 19;14(6):e250108. doi: 10.1530/EC-25-0108 (PMC12186297; doi:10.1530/EC-25-0108)
Supplement: Supplementary file 1 [file supplementary_materials.pdf]

Supplementary Data

Identifying confounders

ICD-10 codes for alcohol overconsumption:

E24.4, F10, G62.1, I42.6, K29.2, G31.2, G71.2, K70, K85.2, K86.0, O35.4, T51.0, T51.9, R78.0, Y57.3, X65, Y90, Y91, Z50.2, Z71.4, Z72.1.

Supplementary Table 1. Risk estimates of the confounders on breast cancer risk using multivariate cox regression analysis.

Variable	Hazard Ratio (95% Confidence interval)
Diabetes mellitus	1.595 (1.135-2.241)
Obesity	1.214 (0.541-2.725)
Smoking/COPD	1.383 (0.617-3.098)
Alcohol overconsumption	0.289 (0.039-2.124)
HRT prescription	1.199 (0.912-1.576)
Educational level	
- 0-9 years	1.00
- 10-12 years	0.657 (0.453-0.953)
- >12 years	0.609 (0.418-0.886)

Abbreviations: COPD, chronic obstructive pulmonary disease; HRT, hormone replacement therapy.
ICD-10 codes: Diabetes mellitus, E10-14; Obesity, E66; Smoking, F17, Z71.6, Z72; Chronic obstructive pulmonary disease, J43-44; Alcohol overconsumption E24.4, F10, G62.1, I42.6, K29.2, G31.2, G71.2, K70, K85.2, K86.0, O35.4, T51.0, T51.9, R78.0, Y57.3, X65, Y90-91, Z50.2, Z71.4, Z72.1. ATC-codes: HRT prescription, G03C, G03D, G03F, G03XC, G02BA03.
